# Effects of Heart Failure Therapies on Atrial Fibrillation: Biological and Clinical Perspectives

**DOI:** 10.3390/antiox13070806

**Published:** 2024-07-02

**Authors:** Alfredo Mauriello, Antonia Ascrizzi, Anna Selvaggia Roma, Riccardo Molinari, Alfredo Caturano, Egidio Imbalzano, Antonello D’Andrea, Vincenzo Russo

**Affiliations:** 1 Cardiology Unit, Department of Medical and Translational Sciences, University of Campania “Luigi Vanvitelli”, Monaldi Hospital, 80131 Naples, Italy; alfredo.mauriello93@libero.it (A.M.); antonia.ascrizzi@studenti.unicampania.it (A.A.); annaselvaggia.roma@studenti.unicampania.it (A.S.R.); riccardo.molinari@studenti.unicampania.it (R.M.); 2 Cardiology and Intensive Care Unit, Department of Cardiology, Umberto I Hospital, 84014 Nocera Inferiore, Italy; antonellodandrea@libero.it; 3 Department of Clinical and Experimental Medicine, University of Messina, 98100 Messina, Italy; alfredo.caturano@unicampania.it; 4 Department of Advanced Medical and Surgical Sciences, University of Campania “Luigi Vanvitelli”, 80131 Naples, Italy; egidio.imbalzano@unime.it

**Keywords:** atrial fibrillation, heart failure, empagliflozin, dapagliflozin, vericiguat, sacubitril/valsartan

## Abstract

Heart failure (HF) and atrial fibrillation (AF) are prevalent cardiovascular diseases that contribute significantly to morbidity, mortality, hospitalisation, and healthcare costs. It is not uncommon for these conditions to coexist and have mutually reinforcing effects. A critical factor in the aetiology of these conditions is oxidative stress, driven by reactive oxygen species (ROS), which contributes to atrial remodelling and fibrosis. The recent introduction of new drugs for the treatment of heart failure has also had an impact on the management of atrial fibrillation due to their influence on oxidative stress. The objective of this review is to analyse the effects of these therapies, including their role in mitigating ROS, on the prevention and treatment of AF in HF patients.

## 1. Introduction

**Figure 1:**
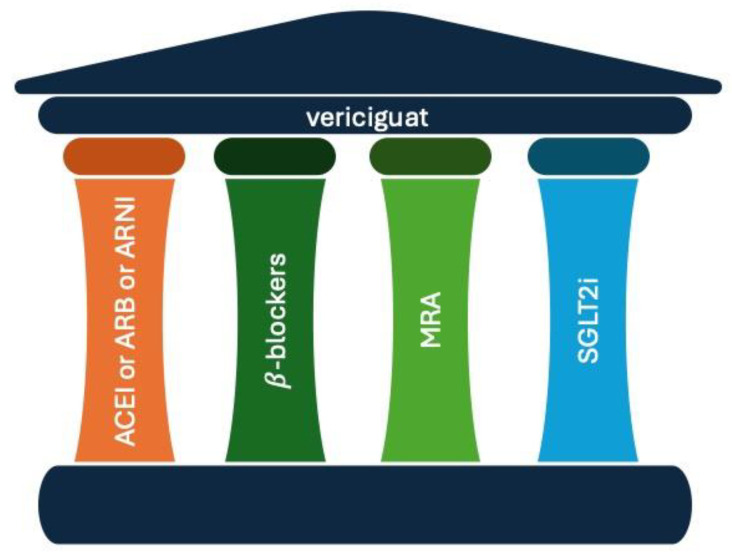
The five pillars of therapy for the pharmacological treatment of chronic heart failure. ACEI: angiotensin-converting enzyme inhibitors; ARNI: angiotensin receptor neprilysin inhibitors; ARB: angiotensin receptor blocker; MRA: mineralocorticoid receptor antagonist; SGLT2i: sodium-glucose transport protein inhibitors.

Heart failure (HF) is a clinical syndrome characterised by typical symptoms (e.g., breathlessness, ankle swelling, and fatigue) that may be accompanied by signs (e.g., elevated jugular venous pressure, pulmonary crackles, and peripheral oedema) caused by a structural and/or functional cardiac abnormality, resulting in a reduced cardiac output and/or elevated intracardiac pressures at rest or during stress [1]. In Europe, the HF incidence is about 3–5/1000 person-years. The HF prevalence is about 1–2% in adults and it increases with age, accounting for up to 10% in patients aged 70 years or over [1]; however, considering the new European Society Cardiology (ESC) classification, which includes HF with preserved (HFpEF) and mid-range (HFmrEF) ejection fractions, an increased prevalence of about 50% worldwide is expected [2]. According to the ESC Long-Term Registry, which includes 12,440 patients in an outpatient setting, the prevalence of HFpEF, HFmrEF, and HFrEF was 60%, 24%, and 16%, respectively. Additionally, slightly over 50% of HF patients were women [1]. The prevalence of atrial fibrillation (AF) is increasing worldwide due to increased life expectancy. AF and HF commonly coexist in up to 50% of patients [3]. Among patients with HF, AF is more prevalent in those with HFpEF [3]. In the Swedish HF Registry, which included 76,453 patients, AF prevalence was 50% for HFrEF, 60% for HFmrEF, and 65% for HFpEF [4], and it was associated with greater symptom burdens and worse outcomes, including hospitalisation and mortality [2]. The historical milestone of HF therapy is the combination of angiotensin-converting enzyme inhibitors (ACEI) or angiotensin receptor blockers (ARBs), beta blockers, and a mineralocorticoid receptor antagonist (MRA). Recently, novel pharmacological treatments were introduced, such as sodium-glucose cotransporter 2 inhibitors (SGLT2i), the angiotensin receptor neprilysin inhibitor (ARNI), and vericiguat (Figure 1). 

## 2. AF Electrogenesis in HF

**Figure 2:**
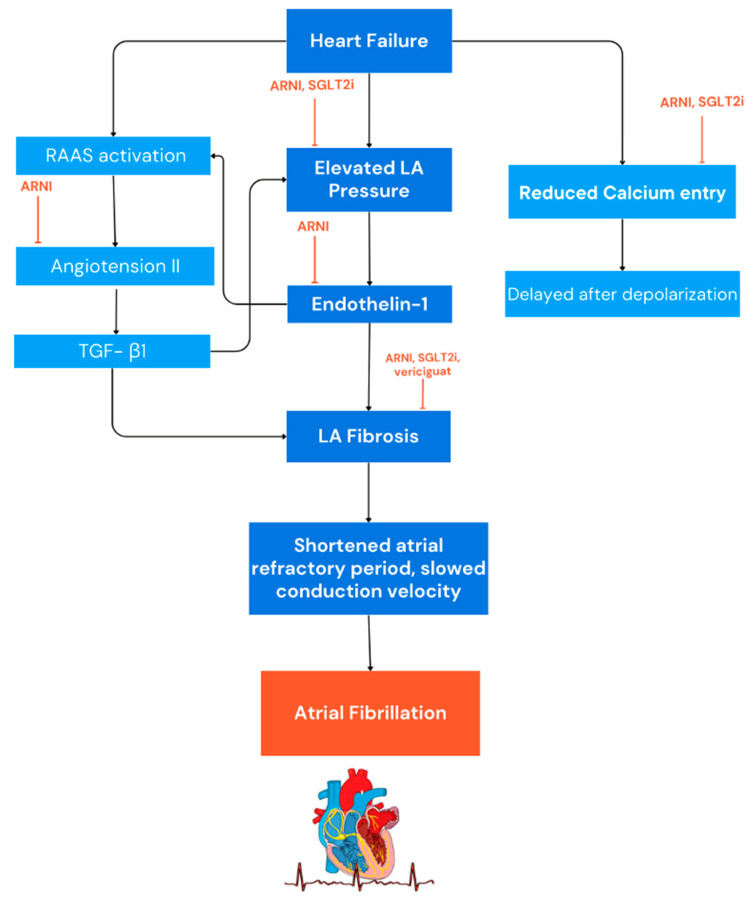
Mechanisms of atrial fibrillation in heart failure. RAAS: renin-angiotensin-aldosterone system; LA: left atrium; ARNI: angiotensin receptor neprilysin inhibitor; SGLT2i: sodium-glucose cotransporter 2 inhibitors.

The pathophysiology of AF in HF is the result of the complex interplay of several mechanisms which contribute to the initiation, maintenance, and progression of arrhythmia (Figure 2). Understanding the deep interaction between AF and HF is of pivotal importance for the optimal management of patients with HF. Several mechanisms may lead to AF in the clinical setting of HF. Several factors, including neurohormonal activation, cardiac stretch, oxidative stress, and endothelial dysfunction lead to an inflammatory state in heart failure [5,6]. The chronic inflammation in patients with heart failure, characterised by elevated levels of pro-inflammatory molecules such as interleukin-6 (IL-6), tumour necrosis factor-alpha (TNF-α), and C-reactive protein (CRP), triggers atrial fibrosis, enlargement, and cell death, disrupting the normal organisation and function of atrial tissue and leading to the development and maintenance of AF. The inflammation state interferes with calcium regulation, ion channel function, and the balance of autonomic nervous system activity, increasing the likelihood of atrial rhythm disturbances and re-entry circuits [7]. In addition, the inflammatory molecules alter the function of ion channels in the atria and the expression of connexins, contributing to irregular atrial conduction and increasing susceptibility to AF triggers [8]. 

In addition to the inflammatory state, recent animal studies have suggested a link between ischaemic events and structural remodelling [9,10,11,12]; moreover, chronic atrial stretch and several neurohormonal abnormalities associated with congestive heart failure (CHF) are likely to play an important role in the development of atrial fibrosis [13]. Atrial stretch induces changes in cellular gene expression, in part mediated by stretch-activated channels, leading to cellular hypertrophy, alterations in ionic transmembrane currents, action potential duration, and increased angiotensin II synthesis [13]. In addition, the activation of neurohormonal pathways, such as the renin-angiotensin-aldosterone system in CHF, is a potent trigger of fibrotic processes [14]. The atrial substrate in HF undergoes profound structural changes, characterised not only by atrial enlargement but also by evidence of the loss of a functional atrial myocardium, with regions with a low voltage amplitude and spontaneous scarring [14]. Histopathological studies show interstitial fibrosis, cellular hypertrophy, and degeneration within the atrial myocardium, promoting conduction abnormalities and re-entrant circuits [13,15,16,17]. Regions of low voltage and fractionated signals reflect slowed conduction and conduction delay, setting the stage for arrhythmogenesis [14]. In contrast to the classical “AF begets AF” paradigm, RF-induced electrophysiological changes contribute to AF susceptibility through complex mechanisms [9]. An animal study showed an increased atrial effective refractory period (ERP) and conduction heterogeneity, despite the absence of ERP shortening [9]. Atrial electrical remodelling in HF involves discrete regions of slow conduction associated with interstitial fibrosis, facilitating AF initiation and perpetuation [9,18]. Chronic atrial stretch and neurohormonal abnormalities in HF induce sinus node dysfunction, potentially predisposing patients to bradycardia-dependent AF [13]. 

Natriuretic peptides, including atrial natriuretic peptide (ANP) and brain natriuretic peptide (BNP), exhibit intricate functions in cardiac physiology [19]. ANP and BNP, released in response to cardiac stretch, play key roles in regulating fluid balance and vascular tone [20]. These peptides exert anti-fibrotic, anti-hypertrophic, and vasodilatory effects that counteract adverse remodelling processes in HF [20,21,22]. Some studies have indicated that these peptides may contribute to structural remodelling in the atria, potentially facilitating the formation of arrhythmogenic substrates [19,23]. At the cellular level, ANP and BNP modulate intracellular calcium handling, ion channel function, and fibroblast activity, thereby influencing atrial electrophysiology and remodelling [20]. They promote atrial dilatation, fibrosis, and electrical remodelling, creating a substrate favourable to the maintenance of AF [24]. This effect appears to be context-dependent and may contrast with the protective effects observed in clinical studies with neprilysin inhibitors, which elevate natriuretic peptide levels. It is noteworthy that sacubitrilat also increases the levels of the C-type natriuretic peptide (CNP), which has been demonstrated to possess antiarrhythmic properties, at least in ventricular cells [19,23]. Further research is required to fully understand the differential impacts of these peptides on atrial versus ventricular remodelling.

In HF, changes in atrial calcium handling are similar to those observed in AF [25]. In atrial fibrillation, there is a well-documented reduction in the L-type Ca^2+^ current, which contributes to the electrical remodelling observed in this condition. Primary studies have demonstrated that, in atrial fibrillation (AF), the density of the L-type Ca^2+^ current is significantly reduced, resulting in a shortened action potential duration and altered calcium handling in atrial myocytes [26,27]. These changes contribute to the maintenance of the arrhythmogenic substrate characteristic of AF [25,28]. In addition, HF is characterised by an increase in sarcoplasmic reticulum (SR) calcium content, similar to findings in early or paroxysmal AF, but is not consistently observed in persistent AF [25]. Although data on the effects of HF on atrial ryanodine receptors (RyRs) are limited, it appears that HF does not induce RyR phosphorylation [25]. However, both HF and AF contribute to increased RyR leakage and after depolarisation, thereby promoting arrhythmogenesis [25].

## 3. Oxidative Stress and Atrial Fibrillation 

Oxidative stress, which reflects an imbalance between the production of reactive oxygen species (ROS) and the ability of a biological antioxidant system to detoxify these reactive intermediates or repair the resulting damage, plays a central role in several cardiovascular diseases, including AF and HF [29]. This imbalance leads to cellular damage, inflammation, and fibrosis, which are critical mechanisms in the development and maintenance of AF and the progression of HF [30,31,32].

Several studies have implicated increased oxidative stress within the atrial tissue in the development of AF, suggesting a significant role in the remodelling phenomenon [30,31,32]. Mihm et al. were among the first to investigate the energetic status of myofibrils and the oxidative modification of proteins in right atrial appendage biopsies from patients with chronic AF [30]. They found increased nitrotyrosine and protein carbonyl formation, suggesting that oxidative stress plays an important role in this setting [30]. Further experimental evidence suggests that atrial tachycardia induces calcium accumulation, which leads to increased oxidative stress and a significant decrease in atrial tissue vitamin C levels, while also being associated with increased protein nitration [33,34,35].

Atrial tissue from patients with atrial fibrillation has low levels of glutathione, an abnormality associated with downregulation of the L-type calcium current due to S-nitrosylation [33]. Recent studies examining remodelling in atrial biopsies from patients with persistent atrial fibrillation have shown increased atrial tissue levels of oxygenase-1 and 3-nitrotyrosine, together with cardiomyocyte hypertrophy, myolytic damage, and interstitial fibrosis [36]. These findings suggest that atrial oxidative damage and associated structural remodelling occur early after the clinical onset of AF and not solely due to underlying cardiovascular disease [36]. Oxidative stress is more pronounced in the setting of atrial fibrillation following cardiac surgery where tissue damage and ischaemia-reperfusion injury occur [37,38]. Several sources of ROS in AF have been reported, including NADPH oxidase, xanthine oxidase, nitric oxide synthase uncoupling, mitochondrial dysfunction, myeloperoxidase, and monoamine oxidase [29]. Among these, NADPH oxidase is a major contributor to atrial oxidative stress [29]. Associated conditions, such as hyperglycaemia, hyperlipidaemia, hypertension, and elevated plasma fatty acid levels, can increase angiotensin II levels, leading to the activation of NADPH oxidase [29]. Studies have shown that myocardial NADPH oxidase and dysfunctional NOS contribute to superoxide generation and oxidative damage in human atrial tissue in the setting of AF [39].

Mitochondrial dysfunction leading to mitochondrial ROS production has been implicated in ryanodine receptor oxidation, facilitating calcium leakage and AF development [40]. Myeloperoxidase (MPO), an enzyme released by activated polymorphonuclear neutrophils, has been implicated in atrial fibrosis and remodelling, promoting the activation of pro-metalloproteinases and the deposition of atrial collagen, leading to atrial arrhythmias [41,42].

Several biomarkers of oxidative stress have been associated with the development, persistence, burden, and severity of AF, as well as with AF recurrence [29]. However, the cause-effect relationship between oxidative stress biomarkers and AF remains unclear, although data suggest a pathophysiological link and a potential predictive role in AF management [29]. Biomarkers such as uric acid and γ-glutamyl transferase (γGT) may indicate oxidative stress in this setting [43]. Elevated levels of these markers have been associated with the risk of AF and AF recurrence after ablation [44,45,46,47]. Medications used to treat heart failure often have antioxidant properties that can reduce the harmful effects of oxidative stress. Angiotensin-converting enzyme inhibitors, angiotensin II receptor blockers, beta blockers, and aldosterone antagonists have been shown to reduce oxidative stress markers and improve clinical outcomes in HF patients [48]. These drugs help to preserve myocardial function, reduce fibrosis, and improve overall cardiac performance by reducing oxidative stress [48]. This review aims to explore the role of oxidative stress as a modulator of AF and examine the antioxidant effects of drugs used to treat heart failure. Understanding these mechanisms may provide insights into the complex interplay between oxidative stress and cardiovascular disease, potentially leading to more targeted and effective therapeutic strategies. By elucidating the antioxidant properties of heart failure drugs, this review aims to highlight their dual role in managing both HF and the oxidative stress-related components of atrial fibrillation, ultimately contributing to better patient outcomes.

## 4. Historical Use of Digitalis and NO Donors in HF Management

Digitalis has a well-documented history of use in the treatment of HF and AF. Historically, digitalis was primarily evaluated for its impact on mortality and symptomatic improvement, without the composite endpoints that are used in modern trials. The Digitalis Investigation Group (DIG) study, for instance, focused solely on mortality and hospitalisation for heart failure, rather than combining various clinical outcomes [49]. Furthermore, digitalis exerts its effects by inhibiting Na, K-ATPase, which is mechanistically linked to the SGLT2 pathway. This offers a distinct therapeutic action compared to contemporary HF treatments [50].

Similarly, nitric oxide (NO) donors, such as trinitrine, were historically employed for their vasodilatory effects, with the aim of preserving NO levels. The clinical trials that have evaluated these agents did not employ composite outcomes, rather, they focused on specific endpoints, such as mortality and symptomatic relief. Vericiguat, a contemporary NO pathway modulator, has been subjected to clinical trials employing composite outcomes, reflecting the evolution in trial design to capture a broader spectrum of clinical benefits and risks [50].

## 5. Angiotensin Receptor Neprilysin Inhibitors (ARNI)

Angiotensin receptor neprilysin inhibitors (ARNIs) are a class of drugs that combine an angiotensin receptor blocker (ARB) with a neprilysin inhibitor. The pioneering ARNI is sacubitril/valsartan (S/V), which combines valsartan, an angiotensin II type 1 receptor blocker, with sacubitril, a neprilysin inhibitor [51]. 

The Prospective Comparison of ARNI With ACEI to Determine Impact on Global Mortality and Morbidity in Heart Failure (PARADIGM-HF) trial enrolled 8.442 HFrEF patients with a mean age of 63.8 ± 11.5 years, 22% of whom were female [52]. Among the overall study population, 36% had a baseline diagnosis of AF with no difference between the two groups (36.2% in the ARNI group and 37.4% in the enalapril group) [52]. The PARADIGM-HF trial showed that S/V significantly reduced all-cause mortality (−16%) and cardiovascular death (−20%) compared to standard medical therapy [53]. In both the S/V and enalapril groups, approximately 3.1% of patients developed new-onset AF [53]. In a sub-analysis of the PARADIGM-HF trial, a significant 20% decrease in sudden cardiac death (SCD) was shown, regardless of the presence of an implantable cardioverter defibrillator (ICD) [54].

The dual effects of sacubitril/valsartan on oxidative stress are mediated by the ability to reduce angiotensin II levels, which in turn suppresses NADPH oxidase activity, a significant source of ROS in the pathogenesis of atrial fibrillation (AF) [52]. This action has the potential to mitigate oxidative stress in the atria, thereby reducing structural remodelling and the promotion of AF [55]. The antiarrhythmic properties of S/V therapy were further supported by two additional prospective studies which included 151 ICD recipients with HFrEF (<35%) and 120 ICD recipients with HFrEF (≤40%). Both studies showed a significant decrease in non-sustained and sustained ventricular tachycardia episodes, appropriate ICD shocks, and premature ventricular contractions compared to those occurring before the initiation of sacubitril/valsartan. Moreover, there was a noticeable increase in the percentage of biventricular pacing [56,57,58]. 

Currently, few observations describe the effect of ARNI on AF recurrence. In a prospective observational study including 120 ICD recipients with HFrEF (≤40%), de Diego et al. found a trend towards a reduction in paroxysmal atrial tachycardia or AF episodes after S/V during a 9-month follow-up [57]. In a prospective observational study including 167 ICD recipients with HFrEF, Russo et al. [59] showed that S/V treatment was linked to a significant decrease in AF episodes and an improvement in P wave sensing and the atrial pacing threshold during a 12-month follow-up.

Two retrospective studies were conducted to examine the effects of sacubitril/valsartan on individuals undergoing either pharmacological or electrical cardioversion. De Vecchis et al. demonstrated that S/V was associated with a reduced risk of AF recurrence at a one-year follow-up in a cohort of 40 patients with AF and heart failure with HFrEF. The cohort had a mean age of 76 ± 5.5 years, with male predominance (70%) observed [60]. Chen et al. demonstrated, in another retrospective study of 76 patients with persistent AF post-cardioversion, that the use of sacubitril/valsartan can reduce the rate of recurrence after electrical cardioversion (HR 0.35, 0.14–0.91) [61]. Although retrospective studies offer valuable insights, they are often more susceptible to bias than prospective studies and randomised controlled trials (RCTs). This is due to the reliance on pre-existing data and the potential for selection bias. It is therefore recommended that, while they are valuable for hypothesis generation, their findings should be interpreted with caution and ideally confirmed by higher-level evidence.

Furthermore, in a recent randomised trial, treatment with sacubitril/valsartan, administered on the day of RFCA and lasting for a year, has been shown to reduce heart rate and sympathetic tone and be safe and effective in preventing AF recurrences [62].

A prospective study enrolling 40 patients evaluated the impact of catheter ablation on sympathetic nerve function in patients with AF and showed that ablation reduces adrenergic tone in patients with HF and predicts AF recurrence [63,64,65]. 

Sacubitril/valsartan does not have an intrinsic direct antiarrhythmic effect, but its biochemical and mechanical effects may explain the reduction of AF burden. First, it inhibits neprilysin, an enzyme responsible for the degradation of natriuretic peptides, including the atrial natriuretic peptide (ANP) and B-type natriuretic peptide (BNP), allowing these protective hormones to regulate atrial and ventricular function more effectively. At the atrial level, it preserves the reservoir function, which involves the aspiration of caval and pulmonary venous blood into the atria during ventricular systole [60]. Additionally, at the ventricular level, it improves stroke volume by enhancing contractile efficiency, particularly in the sagittal–longitudinal axis [66]. Therefore, S/V can restore haemodynamic balance and normalise cardiac rhythm by inducing both morphological and electroanatomical “reverse remodelling” of the atria, resulting in improved mechanical and electrophysiological properties as well. This could be a strategy to reduce the recurrence of atrial fibrillation after radiofrequency catheter ablation (RFCA) [57]. 

An animal model study by Li et al. demonstrated that S/V reduced the electrical and structural remodelling of AF [67]. Thirty-three rabbits were divided into sham, rapid atrial pacing (RAP), and S/V groups. The study found that the RAP group had a larger atria and right ventricles, more significant myocardial fibrosis, higher AF inducibility, and shorter atrial refractory periods compared to the sham group. However, these effects were significantly reversed by S/V. The RAP group also showed increased levels of collagen and biomarkers such as the N-terminal-pro-brain natriuretic peptide (NT-proBNP) and ST2, as well as increased activity of calcineurin and the nuclear factor of activated T-cells (NFAT), while recombinant NFAT (p-NFAT) and Cav1.2 were reduced. Additionally, it also reversed the increase in Ca^2+^ concentration and decrease in ICaL density observed in the AF models [67]. Further, S/V reduces neurohormonal activation by inhibiting the renin-angiotensin-aldosterone system (RAAS) through the valsartan component. This reduction in neurohormonal activation can contribute to the prevention of atrial remodelling and AF. Angiotensin II has been implicated in atrial fibrosis and electrical remodelling, both of which are involved in the pathogenesis of AF. By reducing preload and afterload, this treatment can alleviate left atrial pressure and volume overload, which are known triggers for AF. On top of that, both sacubitril and valsartan have antifibrotic properties and S/V may decrease the substrate for AF by reducing fibrosis in the atria. Cardiac sympathetic nerve activity has also been implicated in the development and recurrence of AF in several animal studies [68,69]. Table 1 summarises the cited studies.

## 6. Sodium-Glucose Co-Transporter 2 Inhibitors (SGLT2i)

Sodium-glucose co-transporter 2 inhibitors (SGLT2i) are a class of drugs that block the reabsorption of glucose in the early proximal tubule of the nephron, leading to increased glycosuria and reduced glycemia [71]. SGLT2 inhibitors have been shown to reduce the risk of HF hospitalisation and cardiovascular death independently from the glucose-lowering effects [72] and are recommended as the standard therapy for patients with HFrEF, including those without diabetes [73]. RCTs focused on the efficacy of SGLT2i on specific AF-related endpoints are currently lacking. Despite this, a trend towards a reduction of AF burden can be highlighted in some trials [74,75,76], where a statistically significant difference from the placebo group is hardly detected due to the underpowering caused by the small number of incident AF episodes and the relatively short duration of the follow-up. SGLT2 inhibitors exert their effects in AF through multiple mechanisms, including improved glycaemic control and a reduction of cardiovascular risk factors such as hypertension and obesity, which are associated with increased oxidative stress [77]. From a pathophysiological perspective, the pleiotropic properties of SGLT2 inhibitors may have a beneficial influence on the incidence of AF [78,79]. Additionally, these drugs have been shown to reduce oxidative stress markers in cardiovascular tissues. Notable markers include malondialdehyde (MDA), a marker of lipid peroxidation [80], 8-isoprostane [81], nitrotyrosine [82], ROS [82], superoxide [82], oxidised low-density lipoproteins (oxLDLs) [82], and glutathione (GSH) [82], suggesting a direct antioxidant effect that may mitigate oxidative damage in AF [83].

### 6.1. Dapagliflozin

In the “Dapagliflozin in Patients with Heart Failure and Reduced Ejection Fraction” (DAPA-HF) trial, 4744 HFrEF patients were randomised to receive either dapagliflozin or a placebo alongside recommended therapy [84]. 

During a median follow-up of 18.2 months, a 36% reduction in the relative risk of the primary outcome, defined as a combination of worsening heart failure and cardiovascular death, was observed in the dapagliflozin group compared to the placebo group [84]. The event rates for the two components of the composite outcome were in favour of dapagliflozin with a 30% reduction in hospitalisation for heart failure and 18% reduction in cardiovascular death [84]. 

In a post-hoc analysis of DAPA-HF, dapagliflozin showed a similar degree of reduction in the occurrence of the primary outcome in patients with and without AF [32]. Consistent benefits were also observed for the individual components of the primary outcome, all-cause mortality, and improvement in the Kansas City Cardiomyopathy Questionnaire (KCQQ) total symptom score [32] for AF patients. 

Although the occurrence of AF/atrial flutter (AFL) events in the group receiving SGLT2 inhibitors was lower compared to the placebo group, dapagliflozin did not show a significant reduction in the risk of new-onset AF compared to placebo [32].

The Dapagliflozin in Patients with Chronic Kidney Disease (DAPA-CKD) trial randomised 4304 CKD patients to receive dapagliflozin or a placebo [85]. Over a median follow-up of 2.4 years, a 39% reduction in the occurrence of the primary outcome, defined as a composite of a sustained decline in the estimated GFR of at least 50%, end-stage kidney disease, and death from renal or cardiovascular causes, was observed in the dapagliflozin group. Although patients on dapagliflozin experienced fewer serious adverse events of AF compared to the control patients (8 vs. 17), the limited number of incident AF episodes and the short follow-up did not lead to a significant conclusion [85].

The “Dapagliflozin and Cardiovascular Outcomes in Type 2 Diabetes” (Declare TIMI 58) trial evaluated the cardiovascular efficacy and safety of dapagliflozin in 17,160 patients with type 2 diabetes mellitus (DM) and either multiple risk factors for or established atherosclerotic cardiovascular disease (ASCVD) [86]. The study showed a lower rate of cardiovascular death or hospitalisation for HF among patients treated with dapagliflozin compared to the placebo (HR 0.83; *p* = 0.005) [86]. During a median follow-up period of 4.2 years, a total of 769 AF/AFL events occurred in 589 patients [86].

In a post-hoc analysis (DECLARE–TIMI 58 trial), dapagliflozin significantly reduced the risk of a first AF/AFL event during the follow-up by 19% [87] and it led to a 23% decrease in the total number of AF/AFL events [87]; notably, these reductions were consistent across patients with and without a known history of AF/AFL. Moreover, the presence of ASCVD or a history of heart failure did not impact the observed reduction in AF/AFL events among patients randomised to receive dapagliflozin [87].

In the “Dapagliflozin in Heart Failure with Mildly Reduced or Preserved Ejection Fraction” (DELIVER) trial, 6263 patients with heart failure and a left ventricular ejection fraction < 40% were randomised to dapagliflozin or a placebo, in addition to standard therapy [88]. An 18% reduction of the occurrence of primary outcome, defined as a composite of worsening heart failure, was observed in dapagliflozin treatment group, over a median follow-up period of 2.3 years. These results were consistent across prespecified subgroups, including patients with or without DM or with a left ventricular ejection fraction of more or less than 60% [88]. 

In a post-hoc analysis of the DELIVER trial focused on patients with HFimpEF, dapagliflozin was associated with lower rates of cardiovascular death compared to the placebo (HR 0.62, *p* = 0.09); in particular, a significant reduction in sudden cardiac death was reported (HR 0.99), particularly in the HFimpEF group (HR 0.38, *p* = 0.02) [89]. It is interesting to note that the reduction in sudden cardiac death with dapagliflozin was observed regardless of the achieved left ventricular ejection fraction (EF), no matter whether it was equal to or greater than 50% or less than 50% [89].

At the baseline, of the 6,261 patients enrolled in DELIVER, 43.3% had no AF, 18.0% had paroxysmal AF, and 38.7% had persistent/permanent AF [90]. 

Another post-hoc analysis [90] of the DELIVER trial demonstrated that the risk of the primary endpoint was higher in patients with AF, particularly those with paroxysmal AF, driven by a higher rate of heart failure hospitalisation. However, the beneficial effects of dapagliflozin on the primary outcome were consistent across different types of AF. Similar effects were observed for HF hospitalisation, cardiovascular death, all-cause mortality, and improvement in the (KCCQ) symptom score. Regarding the AF incidence during the follow-up, in the DELIVER trial, the occurrence rates of AF events were similar in the treatment and placebo groups (SAEs of AF in treatment group 57 [1.8%] vs. 47 [1.5%] in the placebo group) [88]. 

Recently, the Dapagliflozin in Myocardial Infarction without Diabetes or Heart Failure (DAPA-MI) trial firstly included a specific component for new-onset AF in the composite endpoint and showed a statistically significant low rate of new-onset AF over a median follow-up of 11.6 months in the dapagliflozin group (HR 0.88, 95% CI 0.45–1.73) [91].

### 6.2. Empagliflozin

The “Empagliflozin Outcome Trial in Patients with Chronic Heart Failure with Preserved Ejection Fraction” (EMPEROR-Preserved) trial randomised 5988 patients with HFpEF to receive either empagliflozin or a placebo on top of recommended therapy [92]. During a median follow-up of 26.2 months, a 21% reduction of the relative risk of the primary outcome, defined as a composite of death from cardiovascular causes or hospitalisation for heart failure, was observed in the empagliflozin treatment group compared to the placebo group [92]. Of the 5988 patients enrolled, 52% (3135) had AF at the baseline. 

A predefined secondary analysis of the EMPEROR-Preserved trial compared the effects of empagliflozin versus a placebo on the primary and secondary endpoints stratified by baseline AF [93]. Empagliflozin showed a similar reduction in the occurrence of the primary outcome in patients with and without AF [93]. There was also a similar reduction in the rate of decline in renal function independent of AF. During the follow-up, empagliflozin showed no effect on the incidence of new-onset AF during the study [93]. 

The “Empagliflozin Cardiovascular Outcome Event Trial in Type 2 Diabetes Mellitus Patients” (EMPA-REG OUTCOME) enrolled 7028 patients with type 2 diabetes mellitus (DM) at high cardiovascular risk to receive either 10 mg or 25 mg of empagliflozin or placebo in addition to standard care [77]. During a median follow-up of 3.1 years, a 14% reduction of the relative risk of primary outcome, defined as a composite of death from cardiovascular causes, nonfatal myocardial infarction or nonfatal stroke, was observed in the empagliflozin treatment group compared to the placebo group [77]. 

Empagliflozin was associated with a significantly lower risk of death from cardiovascular causes compared with placebo (HR 0.62; *p* < 0.01), while there were no significant between-group differences in the incidence of myocardial infarction or stroke [77]. 

In a post-hoc analysis of the EMPA-REG OUTCOME trial, empagliflozin reduced HF-related and renal events regardless of the presence or absence of AF at the baseline [94]. In the AF group, the absolute number of prevented events by empagliflozin was greater for cardiovascular death or HF hospitalisation (38.0 vs. 8.8 prevented events per 1000 patient-years), cardiovascular death (25.3 vs. to 6.6 prevented events per 1000 patient-years), and HF hospitalisation (20.2 vs. to 4.2 prevented events per 1000 patient-years), concluding that patients with DM, cardiovascular disease and AF may particularly benefit from the use of empagliflozin [94]. The incidence of new-onset AF during the trial was low, with no relevant differences between the placebo and empagliflozin groups (1.6% vs. 2.3%) [94]. 

### 6.3. Meta-Analyses Evaluating the Impact of SGLT2i on AF Occurrence

Since individual studies are underpowered in detecting the impact of SGLT2i on AF recurrence, more robust data can be obtained from meta-analyses.

Li et al. [95] conducted a meta-analysis of 33 trials involving 66,685 patients receiving SGLT2i (dapagliflozin, canagliflozin, empagliflozin, sotagliflozin, and ertugliflozin) with type 1 or type 2 DM, CKD, or HF (both with a reduced EF, mildly reduced EF, or preserved EF) or a combination of these to investigate the protective effect of SGLT2i on AF or AFL.

A significant reduction in serious adverse events (SAEs) of AF/AFL occurrence (HR 0.83, *p* = 0.01) and a similar reduction in SAEs of AF occurrence (HR 0.81, *p* = 0.01) were showed in the SGLT2i group compared to the placebo group [95]. 

In a subgroup analysis, dapagliflozin showed a 27% reduction in the risk of AF/AFL occurrence, while canagliflozin, empagliflozin, ertuflozin, and sotagliflozin did not show a significant reduction [95].

Pandey AK et al. [75], in a meta-analysis including 31 RCTs with a total of 75,279 participants with type 1 or type 2 DM, CKD, HF (both with reduced EF, mildly reduced EF or preserved EF), or a combination of these, confirmed a lower risk of major AF events with the use of SGLT2i (HR 0.75) with a similar reduction in total AF events with SGLT2i [75].

Zheng et al. [96], in a meta-analysis including 20 RCTs involving 63,604 patients, showed that SGLT2i (dapagliflozin, canagliflozin, empagliflozin, and ertugliflozin) were associated with a significant 18% reduction in the risk of incident AF compared with the control group; however, no significant difference in the risk of stroke was shown between the two groups [96].

A further meta-analysis by Li H.L. et al. [97], including 22 RCTs and a total of 52,115 patients, focused on the effect of SGLT2i on cardiac arrhythmias. The results showed a significant reduction in the risk of AF (RR 0.82, *p* = 0.94) and embolic stroke (RR 0.32, *p* = 0.99) in patients treated with SGLT2i. Although not reaching statistical significance, there was a trend towards a reduced risk of AFL (RR 0.83, *p* = 0.45) and cardiac arrest (RR 0.83, *p* = 0.44) [97].

In a meta-analysis including 6 randomised controlled trials and 9467 patients with HFrEF, Sfairopoulos et al. [98], demonstrated a significant reduction in the risk of AF (RR 0.62, *p* = 0.005) and AF/AFL (RR 0.64, *p* = 0.004) in the SGLT2 group compared to the placebo group. 

Empagliflozin was found to result in a significant reduction in the risk of AF and AF/AFL in patients with HFrEF, whereas dapagliflozin use was not associated with a significant reduction in the risk of AF or AF/AFL [98].

An analysis of the FDA adverse event reporting system, encompassing more than 700,000 adverse events, showed a reduced incidence of AF in diabetic patients treated with SGLT2i compared to other glucose-lowering drugs. These results remained consistent, even after excluding reports that listed insulin or antiarrhythmic drugs as concomitant medications, as well as after excluding reports with indications of concomitant cardiovascular disease or renal disease, suggesting a robust antiarrhythmic effect of SGLT2i [76].

In conclusion, the outcomes of the above presented trials show a beneficial trend toward a reduction of AF burden in HF patients, emphasizing the necessity for broader randomised clinical trials, with specific AF endpoints aimed to selectively investigate prospectively the impact of dapagliflozin/empagliflozin in this clinical scenario. Table 2 summarises the anti-arrhythmic outcomes reported in SGLT2i meta-analyses.

### 6.4. Putative Physio-Pathological Mechanism Explaining the Impact of SGLT2i on AF Occurrence

While direct evidence regarding the physiopathological mechanism explaining the effect of SGLT2i on the incidence of AF may be limited, several putative mechanisms have been proposed by researchers based on existing knowledge. One such mechanism revolves around the metabolic and haemodynamic effects of SGLT2i, particularly in the context of HF and diabetes, which are conditions commonly associated with AF. 

One proposed mechanism for the potential reduction in AF with SGLT2i is their beneficial effects on metabolic parameters and cardiac function. These agents have been shown to promote weight loss, reduce blood pressure, and improve arterial stiffness and endothelial function [98]. They also have natriuretic and diuretic effects, leading to a reduction in plasma volume and the relief of congestion in patients with HF [98]. These metabolic and haemodynamic improvements may contribute to a more favourable atrial substrate, potentially reducing the propensity to initiate and maintain atrial fibrillation. 

In 2021, Rau et al. [99] conducted a prospective, randomised, exploratory pilot study to examine the early and delayed effects of empagliflozin treatment on haemodynamic parameters in 44 patients with type 2 DM. The results demonstrated that empagliflozin treatment had no significant effect on the systemic vascular resistance index (SVRI) compared to the placebo after 1 day, 3 days, and 3 months of treatment. However, it was observed to lead to a rapid and sustained significant improvement of diastolic function. In a subsequent sub-analysis of this study, published in 2023, the focus shifted to the effects of empagliflozin on left atrial strain (LAS) in patients with type 2 DM [100]. LAS values can serve as predictors of AF occurrence and recurrence. After three months of treatment with empagliflozin, there was a significant improvement in left atrial function, as indicated by changes in LAS values. 

Given that left atrial strain (LAS) parameters have been established as valuable predictors of AF in various patient cohorts [100], these findings suggest a hypothesis: SGLT2 inhibitors may mitigate AF and AFL by reducing myocardial remodelling through their influence on left atrial filling pressure and left ventricular end-diastolic pressure (LVEDP) [100].

In addition, SGLT2 inhibitors have been associated with the attenuation of inflammation, oxidative stress, and fibrosis [101]; by reducing these, SGLT2 inhibitors may help to preserve the structural and electrical integrity of the atria, thereby reducing the likelihood of AF development. 

Furthermore, SGLT2 inhibitors have been shown to modulate the RAAS and sympathetic nervous system activity [102], both of which play important roles in cardiac remodelling and arrhythmogenesis [68,69]. By targeting these pathways, SGLT2 inhibitors may exert antiarrhythmic effects and reduce the risk of atrial fibrillation [103].

## 7. Vericiguat

Vericiguat is a soluble guanylate cyclase (sGC) stimulant that acts through a binding site independent of nitric oxide (NO), and it sensitises sGC to endogenous nitric oxide by stabilizing nitric oxide binding to the binding site. HF is associated with an impairment of the synthesis of NO and a decreased activity of sGC [42]. Deficiency in sGC-derived cyclic guanosine monophosphate (cGMP) contributes to myocardial and vascular dysfunction. Vericiguat addresses the deficiency in the NO-sGC-cGMP pathway by directly stimulating soluble guanylate cyclase (sGC), both independently and in synergy with nitric oxide (NO), in order to elevate intracellular cGMP levels. This enhancement can improve myocardial function and overall cardiac performance [104]. By promoting NO-mediated vasodilation and reducing myocardial oxygen demand, vericiguat potentially attenuates oxidative stress associated with AF and HF, thus protecting against myocardial damage [104].

The “Vericiguat Global Study in Subjects With Heart Failure With Reduced Ejection Fraction” (VICTORIA) trial randomised 6857 patients with HFrEF (<45%) to receive either vericiguat or a placebo in addition to guideline-based medical therapy [105]. During a median follow-up of 10.8 months, a 18% reduction of the relative risk of primary outcome, defined as a composite of death from cardiovascular causes or first hospitalisation for heart failure, was observed in the vericiguat treatment group compared to the placebo group [104]. Of the 6857 patients enrolled, 45% reported a history of AF at the baseline.

An insight analysis from the VICTORIA trial has evaluated the relation between baseline AF and new-onset AF, as well as the impact of vericiguat treatment on the likelihood of new-onset AF in patients with worsening HFrEF [105]. Among the 5050 patients randomised, the analysis of 5010 patients with recorded AF status revealed varying distributions: 53% of patients had no known AF, 20% had a history of AF alone, and 27% exhibited AF on their randomisation electrocardiogram [105]. Notably, patients with a history of AF alone had had a 21% higher risk of cardiovascular death compared to those without AF [105]. However, the positive effect of vericiguat on the primary composite outcome and its components remained consistent, regardless of AF status at the baseline [105]. Over a median follow-up duration of 10.8 months, new-onset AF emerged in 6.1% of individuals without prior AF and in 18.3% of those with a history of AF alone [105]. Notably, vericiguat treatment did not influence the occurrence of these events (adjusted HR 0.93). Nevertheless, the onset of new AF was associated with an increase in the hazard of both primary and secondary outcomes [105].

In a study involving 36 rabbits, Lou et al. [106] investigated the effect of vericiguat on the induction of AF by rapid atrial pacing (RAP). According to their results, vericiguat effectively reduced the incidence of AF by restoring normal calcium levels, regulating L-type calcium channel currents (ICaL), and maintaining atrial effective refractory periods (AERPs) [106]. These beneficial effects were associated with modulation of the TRPC6/CaN/NFAT pathway, which plays a central role in atrial electrical remodelling. In addition, vericiguat attenuated structural changes by reducing atrial enlargement and fibrosis [73]. Echocardiographic assessment showed a reduction in atrial dimensions and improvement in left ventricular function following the administration of vericiguat [106]. Histological examination confirmed these effects, showing reduced collagen deposition and markers of fibrosis in atrial tissue from vericiguat-treated rabbits, indicating a reversal of structural remodelling [106].

In conclusion, these preliminary studies suggests that vericiguat may represent a novel therapeutic approach for AF by targeting both electrical and structural remodelling in patients with HF. However, further studies are needed to evaluate the effectiveness of vericiguat on AF, and, above all, on clinical outcomes.

## 8. Conclusions

The role of ROS in AF treatments is of significant importance, as oxidative stress is a key contributor to the pathophysiology of both AF and HF. Pharmacological therapies for heart failure do not have direct antiarrhythmic properties, however, their action on RAAS, natriuretic peptides, inflammatory pathways, and intracellular calcium handling lead to reduced atrial fibrosis, inflammation, and abnormal electrical activity, thus potentially attenuating the occurrence and progression of atrial fibrillation. The findings indicate that drugs such as sacubitril/valsartan, SGLT2 inhibitors, and vericiguat can mitigate oxidative stress, thereby offering protective effects against myocardial damage and the development of AF. Future research should focus on comprehensively assessing the efficacy of HF therapies in mitigating AF and improving overall clinical outcomes. Further investigation into the antioxidant properties of these treatments could provide deeper insights into their potential as dual-action therapies, addressing both HF and the oxidative stress-related components of AF. 

## Figures and Tables

**Table 1:** Summary of studies showing anti-arrhythmic effects of ARNIs. AF: atrial fibrillation; CHF: congestive heart failure; ECV: electrical cardioversion; EF: ejection fraction; HFrEF: heart failure with reduced ejection fraction; PALS: peak longitudinal strain; RCT: randomised controlled trial.

Authors	Study Design	Patients, n	Clinical Setting	Outcomes	Follow-Up	Results
Wang et al. [62]	Prospective, RCT	76	HF with EF > 30%	AF recurrence rate	12 months	21.7% *p* < 0.001
Russo et al. [59]	Prospective	167	HFrEF	Reduction in AF episodes	12 months	−9%; *p* = 0.03
Zang et al. [70]	Prospective	140	CHF	Recurrence rate of AF in HF patients	12 months	30%; *p* = 0.005
De Vecchis et al. [60]	Retrospective	40	CHF	Improving PALS	12 months	26.5%; *p* < 0.001
				Decreased risk of AF relapses, n (%)		87.5%; *p* = 0.001
Chen et al. [61]	Retrospective	76	CHF	Ineffective ECV, n (%)	30 days	25%; *p* = 0.02

**Table 2:** Summary of anti-arrhythmic outcomes reported in SGLT2i meta-analyses. AF: atrial fibrillation; AFL: atrial flutter; CKD: chronic kidney disease; CVD: cardiovascular disease; DM: diabetes mellitus; HF: heart failure; HFpEF: heart failure preserved ejection fraction; HFrEF: heart failure reduced ejection fraction; T1DM: type 1 diabetes mellitus; T2DM: type 2 diabetes mellitus; VT: ventricular tachycardia.

Authors	Enrolled Patients, n	Characteristics	SGLT2i	Outcomes	Results
Li et al. [95]	66,685	T2DM, T1DM, CKD, HFrEF, HFpEF, or a combination	Dapagliflozin	Incidence of AF	−29% *p* = 0.003
			Canagliflozin		−20% *p* = 0.19
			Empagliflozin		+15% *p* = 0.59
			Sotagliflozin		−49% *p* = 0.63
			Ertugliflozin		+8% *p* = 0.76
Pandey et al. [75]	75,279	DM, CKD, CVD, HFrEF	Dapagliflozin Canagliflozin Empagliflozin Sotagliflozin Ertugliflozin	SAEs AF/AFL	−25% *p* < 0.0001
				Composite of HF hospitalisation/urgent visit or cardiovascular death in patients with AF at baseline	−30% *p* = 0.0005
Li H et al. [97]	52,115	DM, CKD, HF	Dapagliflozin Canagliflozin Empagliflozin Ertugliflozin	Incidence of AF	−18% *p* hetero
				Incidence of embolic stroke	−68% *p* hetero
				Incidence of AFL	−17% *p* hetero
				Incidence of cardiac arrest	−17% *p* hetero
				Incidence of AF/AFL	−18% *p* hetero
				Incidence of VT	−27% *p* hetero
Zheng et al. [96]	63,604	T2DM, HFrEF, CKD, CVD	Dapagliflozin Canagliflozin Empagliflozin Ertugliflozin	Incidence of AF	−18% *p* = 0.002
				Incidence of stroke	−1% *p* = 0.877
Sfairopoulos et al. [98]	9467	HFrEF	Dapagliflozin	Incidence of AF	−31% *p* = 0.12
				Incidence of AF/AFL	−18% *p* = 0.38
			Empagliflozin	Incidence of AF	−45% *p* = 0.01
				Incidence of AF/AFL	−50% *p* = 0.002

## Data Availability

Not applicable.
